# Endovascular Interventions for the Morbidly Adherent Placenta

**DOI:** 10.3390/jcm7050092

**Published:** 2018-05-01

**Authors:** Claire Kaufman, Anthony Tadros

**Affiliations:** 1 Department of Radiology, University of Utah, Salt Lake City, UT 84112, USA; 2 Department of Radiology, University of California San Diego, San Diego, CA 92103, USA; atadros@ucsd.edu

**Keywords:** placenta accreta, placenta increta, placenta percreta, internal iliac balloon, uterine artery embolization

## Abstract

Morbidly adherent placentas are a spectrum of abnormalities ranging from placental invasion of the myometrium to invasion past the myometrium and muscular layers into adjacent structures. This entity is becoming more prevalent recently with increased number of cesarean deliveries. Given the high risk of morbidity and mortality, this was traditionally treated with pre-term planned cesarean hysterectomy. However, recently, uterine preservation techniques have been implemented for those women wishing to preserve future fertility or their uterus. Early identification is crucial as studies have shown better outcomes for women treated at tertiary care facilities by a dedicated multidisciplinary team. Interventional radiologists are frequently included in the care of these patients as there are several different endovascular techniques which can be implemented to decrease morbidity in these patients both in conjunction with cesarean hysterectomy and in the setting of uterine preservation. This article will review the spectrum of morbidly adherent placentas, imaging, as well as the surgical and endovascular interventions implemented in the care of these complex patients.

## 1. Introduction

In a normal pregnancy, the placental chorionic villi are separated from the maternal myometrium by the decidua basalis. The decidua basalis allows for shearing during labor with contraction of the uterus resulting in separation of the placenta from the uterus [1]. Abnormal placental implantation is caused by invasion of the chorionic villi through the decidua basalis layer into the myometrium [2,3]. While often referred to as placenta accreta, abnormal placental implantation or morbidly adherent placenta, reflects a spectrum of disorders. Placenta accreta occurs when the chorionic villi attach to the uterine myometrium without attachment to the muscle [4]. Placenta increta refers to partial invasion of the chorionic villi into the muscle of the myometrium. Placenta percreta is the most severe form and is defined as chorionic villi invasion through the uterine myometrium and into the serosa layer, with potential involvement of surrounding organs such as bladder, bowel, or abdominopelvic musculature [1,5,6,7].

Risk factors for abnormal placental invasion include prior cesarean delivery, uterine instrumentation including curettage, myomectomy or surgery, placenta previa, advanced maternal age, multiparity, uterine anomalies, and history of invasive placenta during previous pregnancies [5,7,8]. It is not surprising that the incidence of abnormal placental invasion has dramatically increased from approximately 1 in 20,000 live births in 1928 [9] to 1:533–1:300 in 2014 [10]. Among risk factors, prior cesarean delivery and placenta previa are known to carry the highest risk. The reported rate of morbidly adherent placentas after cesarean delivery is 3% after the first delivery, however this increases to 40% after the third cesarean delivery [11]. Combined placenta previa and cesarean delivery further increases the risk [1,11,12,13]. A recent study from the United Kingdom in 2012 found that, in women with prior history of placenta previa and prior cesarean delivery, the rate of placental implantation abnormality was as high as 1 in 20 pregnancies [14]. 

Invasive placental abnormalities are associated with high morbidity and mortality to both the mother and fetus. This includes massive hemorrhage at the time of delivery or attempted removal of the placenta, with maternal mortality rates reported at 6–7% [15,16]. Intra-operative blood loss typically is in the range of 3–5 L [17]. This is increasingly more difficult with the increasing number of obese pregnancies which further complicate surgical interventions and deliveries. Prenatal diagnosis is key for the management of placental implantation abnormalities. Appropriate planning and involvement of a multidisciplinary team is necessary to decrease the morbidity and mortality associated with this condition.

## 2. Imaging

**Figure 1:**
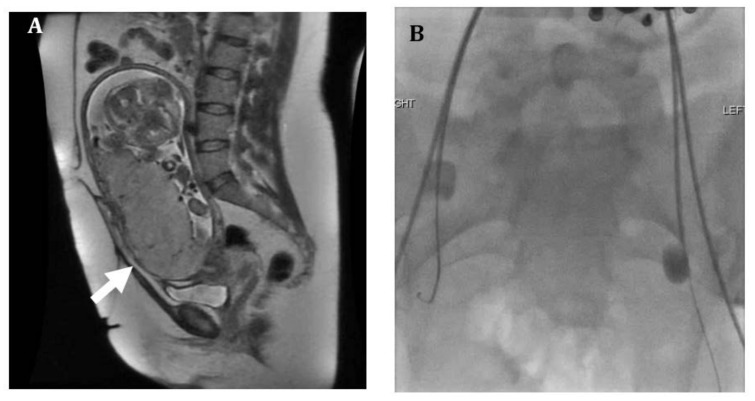
Thirty-three-year-old female with placenta accreta. (**A**) Sagittal SSFSE MR image shows a thickened placenta with abnormal lobulated contour and loss of myometrial thickness in the lower uterine segment (arrow), suggestive of placenta accreta. (**B**) Prophylactic bilateral internal iliac artery occlusion balloons were placed prior to hysterectomy. Patient underwent cesarean hysterectomy at 33 weeks with delivery of a healthy newborn. Pathology showed superficial attachment of the placenta to the uterine wall, consistent with placenta accreta.

**Figure 2:**
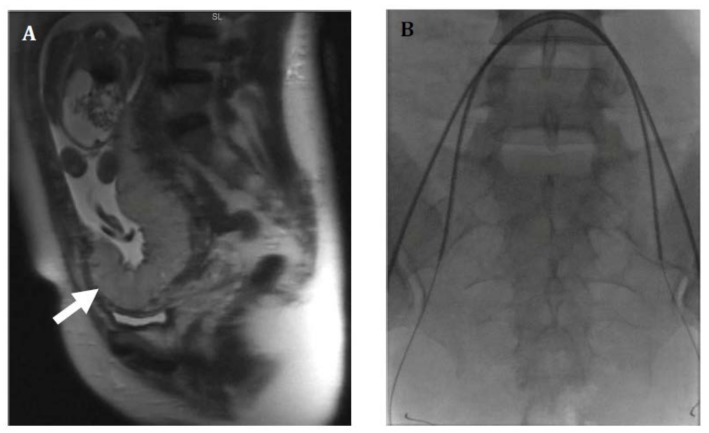
Thirty-six-year-old female with placenta increta. (**A**) Sagittal T2 Half-Fourier-Acquired Single-shot Turbo spin Echo (HASTE) magnetic resonance (MR) image shows a bulging placenta at the lower uterine segment (arrow) with disorganized architecture, suggestive of placenta increta. Complete placental previa as well as an omphalocele were also noted. (**B**) Prophylactic bilateral internal iliac artery occlusion balloons were placed prior to cesarean hysterectomy. Patient underwent cesarean hysterectomy at 22 weeks with delivery of a demised fetus. Patient required extensive lysis of adhesions between the lower uterine segment and bladder.

**Figure 3:**
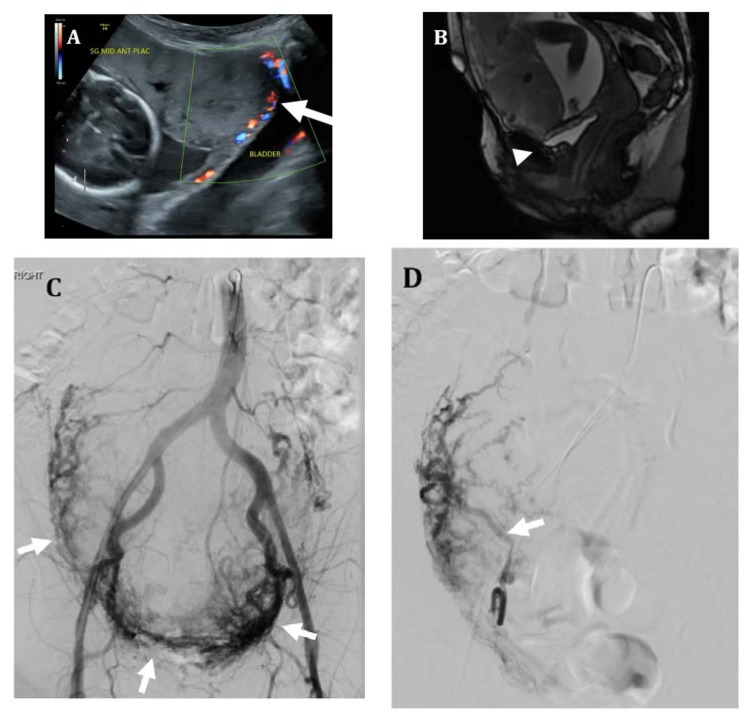

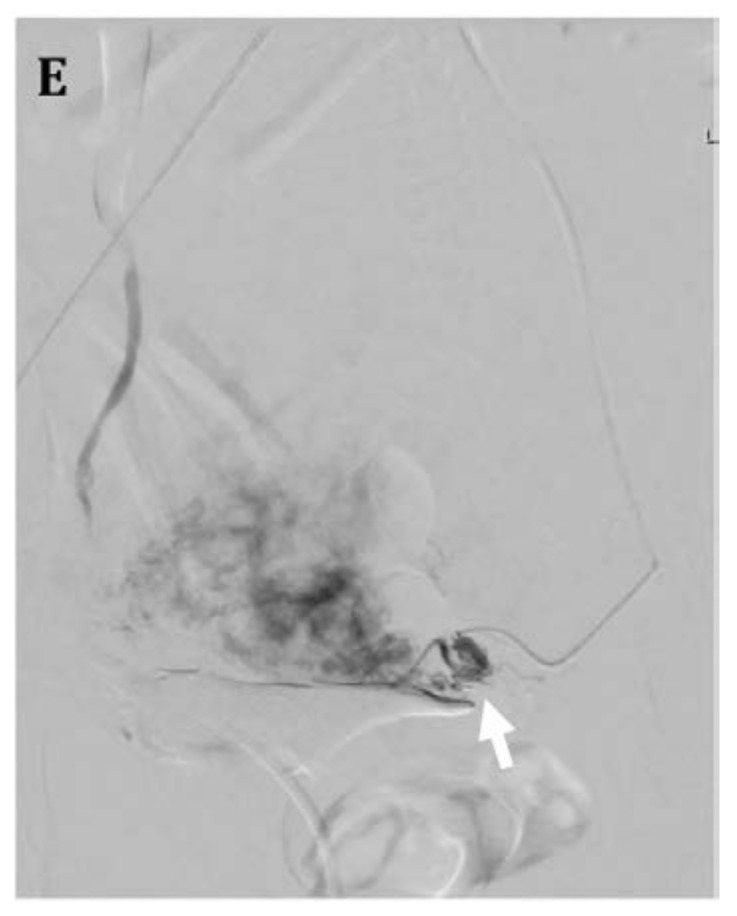
Thirty-five-year-old female with placenta percreta. (**A**) Ultrasound at 19 weeks of gestation shows an abnormal placenta with multiple placental sonolucencies and focal bulging of placental tissue into the bladder (arrow). (**B**) Sagittal FIESTA MR image shows no discernable fat plane between placental tissue and bladder serosa (arrowhead), suspicious for bladder wall invasion. (**C**) Pre-hysterectomy pelvic angiogram shows a large hypervascular placenta (arrows). (**D**) Selective catheterization of the right uterine artery shows an enlarged right uterine artery (arrow) with marked placental hypervascularity. Embolization of bilateral uterine arteries was performed with 500–700-micron polyvinyl alcohol particles until stasis. (**E**) Due to concern for placental invasion of the bladder, the left vesicular artery was also selectively catheterized (arrow) and embolized using 500-micron polyvinyl alcohol particles. Bilateral internal iliac artery occlusion balloons were left in place. Patient ultimately underwent hysterotomy with delivery of a non-viable fetus at 22 weeks; hysterectomy was aborted due to significant pelvic side wall involvement. Patient has been subsequently followed with serial MR imaging for retained placenta.

Placental abnormalities are often first identified using ultrasound on a fetal anatomy scan routinely performed at 18–20 weeks. This typically presents as a low-lying placenta or placenta previa in a patient with a prior history of cesarean delivery. These findings should prompt follow-up imaging later in the pregnancy for further evaluation and planning [18]. Sonographic findings suggestive of placenta accreta include placenta previa, decrease in the thickness of the myometrium, increased vascular flow with lacunae, and loss of the retroplacental clear space [17]. Clear invasion of adjacent structures can sometimes be seen in placenta percreta. Magnetic resonance imaging (MRI) is often used in cases where the patient is high risk but ultrasound was equivocal, or at times to assist in treatment planning [1] (Figure 1 and Figure 2). MRI findings suggestive of an invasive placenta include an irregular border of the placenta that can be nodular or bulging, placenta previa, heterogenous placental parenchyma, and dark irregular intraplacental bands [1,17]. Placenta percreta can further be diagnosed by loss of the normal fat plane between the uterus and adjacent structures (Figure 3) [1,19,20,21,22]. Distinguishing those patients with features of placenta percreta is important as the management and multidisciplinary team involved may vary depending on the imaging findings and involved adjacent structures.

## 3. Surgical Intervention

Once patients with abnormal placental invasion have been identified, the American College of Obstetrics and Gynecology (ACOG) recommends that their care be transferred to a tertiary prenatal center, which has been shown to improve outcomes [23,24]. Many factors weigh in to the timing and planning of the delivery including the extent of placental invasion, and fetal and maternal stability. If possible, delivery is usually scheduled at 34–36 weeks with a multidisciplinary team at a tertiary care facility [15,24,25]. Historically, the recommendation for these patients is to have a planned preterm cesarean hysterectomy. The placenta is left intact and the entire uterus removed after delivery to decrease hemorrhage [23,26]. More recently, conservative treatment that includes uterine preservation has been offered for women who would like to have future children or do not want to lose their uterus. These techniques include expectant management, where the placenta is left in situ after cesarean delivery with no attempt at removal. The mother is subsequently watched closely and over time the placenta resolves. Often the interventional radiologist is involved in these cases to help decrease blood loss [15,27]. Other uterine sparing techniques include hysteroscopic resection of the placenta and en bloc resection of the placenta and myometrium after delivery.

## 4. Endovascular Interventions

Endovascular intervention has been used for both patients undergoing cesarean hysterectomy as well as those who wish to have conservative management with uterine sparing techniques. These techniques often employ the assistance of the interventional radiologist as a key member of the multidisciplinary care team. The techniques range from the use of balloon catheters to embolization.

### 4.1. Internal Iliac Balloon Catheter Placement

Many tertiary care centers employ the use of prophylactic placement of balloon catheters in the internal iliac arteries prior to elective cesarean hysterectomy (Figure 1 and Figure 2). Several studies have shown decrease in intraprocedural blood loss, transfusion requirements, and morbidity during cesarean hysterectomy with the use of internal iliac occlusion balloon catheters [2,28,29,30,31].

While technique may vary depending on institution and operator, prior to the patient undergoing cesarean hysterectomy, bilateral common femoral artery access is usually obtained and sheaths are placed. This is often done simultaneously by two interventional radiologists to minimize procedure time and radiation to the in utero fetus [2,5]. Throughout the procedure, care should be taken to minimize radiation to the fetus by using techniques such as low pulse fluoroscopy rates, fluoroscopy store instead of digital subtraction angiography, collimation, and no magnification. The internal iliac arteries are selected bilaterally from the contralateral approach using the operator’s catheter of choice, often a Cobra 2 catheter (Cook Medical, Bloomington, Indiana). After selection of the anterior division of the internal iliac artery, the catheter is exchanged over the wire for a balloon occlusion catheter with injection of a small amount of contrast to confirm appropriate position. Each balloon catheter should then be tested and the amount required to fill the balloon recorded. The balloons are deflated and the catheters and sheaths are secured. During cesarean section, the balloons are not inflated until after the fetus has been delivered and the umbilical cord clamped. At this time, the balloons can be inflated to help control hemorrhage. Upon conclusion of the procedure, the balloons are deflated and catheters are removed. Often, one sheath is left in place for 24 h in case emergent embolization or resuscitation is required.

The goal of internal iliac artery balloon catheters is to temporarily decrease blood flow to the uterus by decreasing flow to the uterine arteries and other collateral vessels from the anterior division of the internal iliac artery. This aids the surgeons in the removal of the invasive placental tissue or uterus, while decreasing intraoperative blood loss.

### 4.2. Infrarenal Aortic Balloon Catheter

One of the concerns with internal iliac artery balloon occlusion is that it only provides temporary hemostasis. This is due to the extensive collateralization of the pelvic vasculature causing recruitment of other vessels when the internal iliac arteries are occluded. Recently, two studies performed in China reported the use of infrarenal aortic balloon catheter placement prior to cesarean delivery [32,33]. Wu et al. retrospectively examined a population of 230 patients with placenta accreta who underwent prophylactic temporary balloon occlusion of the infrarenal abdominal aorta prior to cesarean delivery. They found significantly decreased blood loss, shorter operative time, decreased transfusion requirement and decreased incidence of hysterectomy in patients who had placement of an infrarenal aortic balloon compared with patients who did not undergo endovascular intervention [32]. Wang et al. performed a prospective study comparing infrarenal aortic balloon occlusion with bilateral internal iliac artery balloon occlusion in patients undergoing cesarean delivery for placenta accreta. They found both procedures to be safe and effective, however the infrarenal aortic balloon occlusion group had shorter fluoroscopy times with therefore decreased radiation dose to the fetus, less intraprocedural blood loss, and shorter overall procedure times [33]. 

In both studies, the abdominal aortic balloon was positioned by an interventional radiologist prior to the procedure from a common femoral artery access. The balloon was inflated after delivery of the fetus to the predetermined size and subsequently verified by lack of pulse oximeter reading. The balloon was left inflated for a set period (5–15 min) followed by 1–2 min of deflation to allow for distal perfusion [32,33].

### 4.3. Uterine Artery Embolization

**Figure 4:**
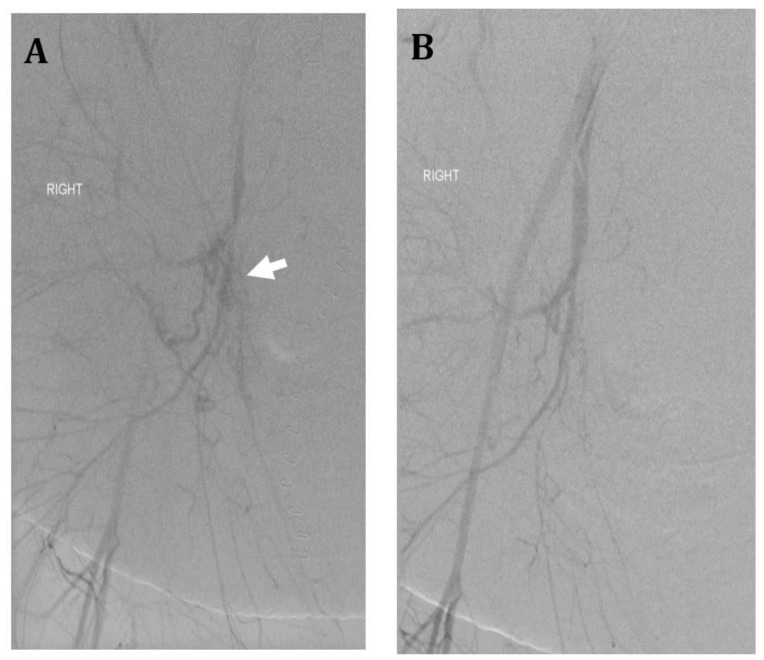
Thirty-six-year-old female with morbid obesity, severe preeclampsia, and placenta accreta status post cesarean section at 34 weeks. Uterine preservation was performed with incomplete removal of placenta. Patient’s postoperative course was complicated by postoperative bleeding and hypotension requiring massive transfusion and vasopressors. (**A**) Right internal iliac artery angiogram shows multiple irregular vessels (arrow). Gelfoam embolization of the anterior division was performed until near hemostasis. (**B**) Post embolization right internal iliac artery angiogram shows no extravasation. Anterior division of left internal iliac artery was also embolized using gelfoam. Despite embolization, patient required hysterectomy for hemorrhagic shock and abdominal compartment syndrome; intraoperative estimated blood loss was approximately 10–15 L. Patient ultimately had a good recovery.

Uterine artery embolization (UAE) is used in two ways for patients with placenta accreta, prophylactically or emergently. UAE can be the sole endovascular treatment or combined with balloon occlusion catheter placement [5]. While traditionally UAE has been used to decrease hemorrhage during combined cesarean hysterectomy, with the implementation of uterine sparing techniques, UAE can now be implemented to assist with placental resorption [15]. Techniques for UAE vary depending on the institutional protocols and published study. While an earlier study described cesarean delivery before endovascular intervention and catheterization of the uterine artery [34], in most instances, common femoral artery access and selective catheterization is obtained prior to cesarean delivery in order to expedite treatment [5,35,36,37]. In some select cases such as known fetal demise or non-viable fetus, UAE may even be performed prior to delivery of the fetus (Figure 3). Depending on the practice, protocols vary from obtaining unilateral to bilateral access as well as selecting the internal iliac arteries versus the uterine artery. Most often, embolization is performed after delivery of the infant to control hemorrhage while decreasing the risk of fetal complication [5,35,36]. One study by Li et al. found that the uterus could be preserved in 10 out of the 12 patients who underwent UAE assisted cesarean delivery for placenta accreta [36]. UAE has been shown to be safe and effective for reducing hemorrhage as well as decreasing rates of hysterectomy in some cases. UAE can also be performed in the post-operative setting in cases of uterine preservation and post-partum hemorrhage (Figure 4).

## 5. Conclusions

Morbidly adherent placentas vary in severity from invasion into the myometrium to invasion of adjacent organs. These abnormalities are associated with high maternal and fetal morbidity and mortality. The ACOG recommends these patients be transferred to a tertiary care facility where they can be managed and treated by a multidisciplinary team. Interventional radiologists play a key role in the management of these patients through a variety of endovascular techniques that can reduce maternal hemorrhage, and, in some cases, assist with uterine preservation for those women wishing to maintain future fertility.
